# Caregiver burden in Parkinson’s disease: a nationwide observational survey

**DOI:** 10.1007/s10072-025-08306-4

**Published:** 2025-06-23

**Authors:** Giulia Donzuso, Paolo Brunelli, Giangi Milesi, Silvia Mancini, Francesca Martillotti, Calogero Edoardo Cicero, Michele Tinazzi, Mario Zappia

**Affiliations:** 1 Department of Medical, Surgical Sciences and Advanced Technologies “GF Ingrassia”, University of Catania, Via Santa Sofia 78, 95123 Catania, Italy; 2 Oasi Research Institute-IRCCS, Troina, Italy; 3 Fondazione LIMPE per il Parkinson ONLUS, Viale Somalia, 133, Roma, 00199 Italy; 4 Confederazione Parkinson Italia, Via Matteo Maria Boiardo 6, Milano, 20123 Italy; 5 Department of Neurosciences, Biomedicine and Movement Sciences, University of Verona, Verona, Italy

**Keywords:** Parkinson’s disease, Caregiver, Quality of life

## Abstract

**Background:**

Caregivers play an important role in Parkinson’s disease (PD), especially in the advanced stages. Aim of this study is to evaluate the caregiver burden of PD in an Italian sample of caregivers.

**Materials and methods:**

An online anonymous survey was conducted among Italian caregivers funded by “Fondazione LIMPE per il Parkinson ONLUS” and “Confederazione Parkinson Italia”. The survey encompassed several dimensions (i.e. caregiving, work, economic and personal health) related to caregivers’ activities and patients’ characteristics.

**Results:**

The survey was completed by 478 caregivers, 361 were women (75%), and the majority had an age included between 55 and 70 years old (46.4%). The burden of assistance increased from 1 to 2 days weekly in the first period of the disease to all the weekly days with the progression of the disease. 15% of caregivers reported not working because of assistance, and among caregivers who were still working, almost 70% reported at least one working day lost monthly due to caregiving activities. Concerning health, most caregivers reported an impact on health due to the assistance, in terms of “excessive tiredness” (74.6%), and “lack of sleep” (60.5%) as the most impacting disturbances. Considering gender, women caregivers reported that they could not work due to the assistance and complained a higher impact on health than men caregivers.

**Conclusion:**

Caregivers of PD patients experienced and reported the presence of caregiver burden in several domains. Additionally, a gender-related pattern was present suggesting the need of a customized support to enhance awareness and minimizing caregiver burden.

**Supplementary Information:**

The online version contains supplementary material available at 10.1007/s10072-025-08306-4.

## Introduction

Parkinson’s Disease (PD) is the second most frequent neurodegenerative disorder [1] and its prevalence is predicted to increase in the near future due to the progressive aging of the general population, reaching up to 14.2 million patients worldwide [2]. PD patients represent a group of chronic patients requiring high costs from the national health services, due to the disability associated with both motor and non-motor symptoms [3]. However, in terms of daily assistance, a major role is played by the caregivers of these patients. Informal caregivers (people who are not financially compensated for providing care, mainly partners or other family members) are usually principal providers of care for patients with PD, representing a major source of underground support for chronic patients. This role can become challenging as the disease progresses, especially given a lack of services and/or perceived lack of coordinated support from health care services, together with the increasing need of patients [4, 5]. Caregiving a PD patient can affect several aspects of a caregiver’s personal life, both in economic and psychological terms, often causing life adjustments for the caregivers that may be forced to leave their jobs to care for their loved ones, not pursue career goals or neglecting family duties to devote time for the relative affected by PD [6]. This condition together with the psychological and well-being consequences that the caregivers experience, is often termed caregiver burden, indicating a multidimensional construct describing “the extent to which caregivers perceived their emotional or physical health, social life, and financial status as suffering as a result of caring for their relative” [7]. Thus, understanding main factors that contribute to burden among informal caregivers of patients with PD is crucial to developing interventions in order to prevent or reduce this burden and support them to maintain well-being and effectiveness in their role [5].

There are several modifiers affecting the burden of caregivers. Concerning gender, women caregivers are usually suffering more from career opportunities withdrawal when compared to men counterparts [8]. Additionally, clinical characteristics of PD patients, such as advanced disease stages, or the severity of non-motor symptoms significantly increases the caregiver burden [5–10]. Some meta-analytic studies deepened the risk factors and the predictors of caregiver burden in caregiver of patients with PD [5, 9], showing that demographics, presence of motor symptoms, motor complications, and general cognitive function did not predict caregiver burden, suggesting that other variables could be predictors of burden, including personal features of caregivers themselves [9]. Additionally, very recently, it has been demonstrated that one of the main factors influencing caregiver burden is the work-life imbalance suggesting that well-being and health of caregivers should be enhanced [5].

In Italy, several studies have been conducted, finding an overall high caregiver burden [11–15]. Interestingly, some studies pointed out how the caregiver burden was associated to PD-related factors [11, 12], whereas other studies evidenced the beneficial effects deriving from therapies also on the caregivers [13, 14]. Indeed, Carpinelli and coll reported the presence of an association between caregiver burden, higher in cohabiting women caregivers of patients with dementia, and the depression/anxiety symptoms, suggesting the need to pay attention to psychological health of caregivers [11]. Considering quality of life, a strong link between caregivers’ well-being and patients’ degree of cognitive impairment was found, with the latter being a strong predictor of caregiver stress and burden [12]. Specifically, Corallo and coll. demonstrated that decrease in patient autonomy regarding basic and instrumental activities of daily living, determined caregivers to exert greater effort in managing patient care [12]. On the other hand, when patients are treated with continuous dopaminergic delivery systems, caregiver burden and quality of life seems to improve [13, 14]. Indeed, caregivers’ partners of advanced PD patients treated with device-aided therapies such as levodopa/carbidopa intestinal gel (LCIG) or continuous subcutaneous apomorphine infusion (CSAI) have more time for their own life and a better perception of their QoL with a tendency to an improvement of mood [13, 14]. However, the different methodologies, the relatively small sample sizes and the differences in patients’ characteristics across the studies limit the generalizability of the results for the Italian PD population.

Considering all these findings, “Fondazione LIMPE per il Parkinson ONLUS”, an Italian foundation associated with the Parkinson Italian Society (LIMPE-DISMOV), with collaboration of “Confederazione Parkinson Italia”, the Italian national network of associations of people with PD, proposed an online survey aiming to define the caregiver burden of PD in Italy. The survey encompassed different domains, including personal and working life aspects, in order to describe the needs and the critical points of caregivers’ activities, allowing to reach a nationwide population of Italian caregivers for person with PD. The questionnaire was developed to explore the needs of caregivers in order to understand how and what kind of initiatives could be developed from “Fondazione LIMPE per il Parkinson ONLUS” to improve caregivers activities. We report here the results of this survey with the aim to disseminate knowledge about the caregiver burden in Italy.

## Materials and methods

### Design

The study had an explorative approach using a semi-structured survey and was carried on following the CHERRIES checklist for web surveys [16]. A key advantage of online qualitative surveys is openness and flexibility to address a wide range of research questions of interest and a suitable research tool with the capacity to deliver rich, deep and complex data for better understanding qualitative data [17]. Furthermore, the ‘anonymous’ mode of responding may also mean participants feel comfortable ‘talking back’ to the researcher [17].

### Ethics and privacy policy

The survey belongs to a group of initiatives promoted by “Fondazione LIMPE per il Parkinson ONLUS” with the aims to support medical-scientific research through fundraising and support for training and the dissemination of information on PD and movement disorders. As an anonymized online survey, ethical approval was not required. The surveys have been conducted anonymously, and consent was implied when respondents acknowledged the privacy policy.

### Study population

Participants were involved with the support of “Fondazione LIMPE per il Parkinson ONLUS” and “Confederazione Parkinson Italia” by advertising all registered patients and caregivers by sending an email with a link to participate in the questionnaire.

### Survey development and administration

The study was conducted using an online anonymized open-survey composed by a semi-structured 39 questions (*n* = 31 single choice questions, *n* = 8 multiple choice questions) dedicated to information related to the caregivers and to information related to the patients, both completed by the caregivers. The questions dedicated to the caregivers consisted of a demographic section and a caregiving features Sect. (12 items), a work Sect. (6 items), an economic Sect. (4 items), and a section dedicated to health and personal impact of the caregiving activities on the caregiver (5 items). The questions dedicated to the patients included socio-demographics and general clinical characteristics of patients (12 items). Domains were selected considering previous studies about predictors of caregiver burden in Parkinson’s disease [5, 9–15, 18, 19]. Furthermore, some caregivers belonging to “Fondazione LIMPE per il Parkinson ONLUS” and “Confederazione Parkinson Italia” have been involved in the development of the survey. In order to develop and test the comprehensibility of the survey, a number of persons belonging to the two promoting associations evaluated the questionnaire prior to dissemination. Data has been gathered using Forms tool on Microsoft 365 (https://www.microsoft.com/it-it/microsoft-365/online-surveys-polls-quizzes). The survey has been conducted from March to April 2024. The survey was sent to all registered patients and caregivers by sending an email with a link to participate in the questionnaire.

### Statistical analysis

Qualitative variables have been described using percentages, and quantitative variables using means and standard deviations. Comparisons have been conducted using Student’s t test or the Mann-Whitney test when appropriate. Categorical variables have been analysed using the chi-squared of Fisher tests. Additionally, a stratified analysis by gender has been performed. Analysis has been conducted using Stata 17.0 (StataCorp. 2023. Stata Statistical Software: Release 17. College Station, TX: StataCorp LLC).

## Results

### Caregivers’ responses

The online survey was promoted by “Fondazione LIMPE per il Parkinson ONLUS” by sending 455 invitation emails. Moreover, “Confederazione Parkinson Italia” invited their associates to participate. The survey was completed by 478 caregivers, but it was not possible to establish the afference of the responders, being the survey anonymized. Not all participants answered to each question (see Table 1 for survey details).

**Table 1:** Socio-demographics characteristics of interviewed caregivers

	Total, *n* = 478
**Gender**,** men (%)**	117 (24.5)
***Age***	*Responders*, *n = 466* *n (%)*
< 25 years	2 (0.4)
26–55 years	104 (22.3)
55–70 years	216 (46.4)
> 70 years	144 (30.9)
***Education***	*Responders*, *n = 466* *n (%)*
Elementary school	15 (3.2)
Middle school	66 (14.2)
High school	214 (45.9)
Graduation	171 (36.7)
***Relationship with patient***	*Responders*, *n = 466* *n (%)*
Partner	340 (72.9)
Son/daughter	90 (19.3)
Brother/sister	10 (2.1)
Friend	3 (0.6)
Other	23 (4.9)
***Proximity to patients***	*Responders*, *n = 462* *n (%)*
Live together	367 (79.4)
Neighbourhood	52 (11.3)
Same city	21 (4.5)
Another city	22 (4.8)
***Duration of assistance***	*Responders*, *n = 423* *n (%)*
< 1 years	28 (6.6)
1–3 years	145 (34.3)
4–10 years	149 (35.2)
> 10 years	101 (23.9)
***Burden of caregiving – first period***	*Responders*, *n = 380* *n (%)*
Occasionally (1–2 days/week)	183 (48.2)
Alternate days	36 (9.5)
Every day	161 (42.4)
***Burden of caregiving – today***	*Responders*, *n = 371* *n (%)*
Occasionally (1–2 days/week)	68 (18.3)
Alternate days	29 (7.8)
Every day	274 (73.8)
***Hourly commitment***	*Responders*, *n = 419* *n (%)*
A couple of hours	149 (35.5)
Half day	92 (22.0)
All day	178 (42.5)
***What kind of assistance***	*Responders*, *n = 455* *(multiple answers given)* *n (%)*
Outside accompaniment	369 (81.1)
Home care	296 (65)
Personal assistance	225 (49.4)
Administration of pharmacological treatment	207 ((45.4)
Supervision	188 (41.3)
***Handling difficult situations***	*Responders*, *n = 456* *n (%)*
Yes	233 (51.1)
No	223 (48.9)
***Training for assistance***	*Responders*, *n = 437* *n (%)*
Yes	41 (9.4)
No	223 (51.0)
No, but it would be useful	173 (39.6)
***What kind of training would you need***	*Responders*, *n = 426* *(multiple answers given)* *n (%)*
None	29 (6.8)
Practical management of assistance activities	205 (48.1)
Information on Clinical Referral Centres	193 (45.3)
Information on home assistance	154 (36.1)
Information on tax breaks	178 (41.8)
Information on legal aspects	223 (52.3)
**Work section**	
***Can you work due to assistance?***	*Responders*, *n = 468* *n (%)*
No	70 (15)
Yes, full time	106 (22.6)
Yes, part time	84 (18)
Retired	208 (44.4)
***Working position***	*Responders*, *n = 180* *n (%)*
Employee	122 (67.8)
Freelance	37 (20.5)
Worker	11 (6.1)
Retired	3 (1.6)
Other	7 (3.9)
***Working days lost monthly***	*Responders*, *n = 179* *n (%)*
0	54 (30.2)
1 day	24 (13.4)
2–5 days	71 (39.7)
6–15 days	15 (8.4)
> 15 days	15 (8.4)
***Presence of working concession/facilitation***	*Responders*, *n = 179* *n (%)*
Yes	97 (54.2)
No	74 (41.3)
Other	8 (4.5)
***Impact on work***	*Responders*, *n = 185* *(multiple answers given)* *n (%)*
None	44 (23.8)
Reduce working hours	58 (31.3)
More working rest days	39 (21.1)
More smart working time	30 (16.2)
Less responsibility role	33 (17.8)
Leave job for a while	14 (7.5)
Change job	14 (7.5)
Job closer to home	9 (4.9)
***Reason of impact on work***	*Responders*, *n = 180* *(multiple answers given)* *n (%)*
No particular reasons	27 (15.0)
Medical reasons	88 (48.9)
Lack of support	8 (4.4)
Need to provide direct assistance	77 (42.8)
Psychological and emotional stress	86 (47.8)
Other	4 (2.2)
**Economic section**	
***Economic support***	*Responders*, *n = 439* *n (%)*
Yes	63 (14.4)
No, we cannot ask for	297 (67.6)
No, but we asked for	79 (18)
***What kind of economic support?***	*Responders*, *n = 61* *n (%)*
Attendance allowance/law 104	47 (77.0)
Other	14 (23.0)
***Economic changes due to caregiving***	*Responders*, *n = 457* *n (%)*
No	184 (40.3)
Yes	273 (59.7)
***Possible causes for economic changes***	*Responders*, *n = 271* *(multiple answers given)* *n (%)*
Rehabilitation treatment	168 (62.0)
Help for house managing	145 (53.5)
Presence of another caregiver	134 (49.4)
Travel/medical visits	112 (41.3)
Accessibility at home	107 (39.5)
Pharmacological treatment	109 (40.2)
Other treatments	81 (29.9)
Other	6 (2.2)
**Health and Personal section**	
***Impact on your health due to caregiving***	*Responders*, *n = 453* *n (%)*
Yes	292 (64.5)
No	161 (35.5)
***What kind of consequences***	*Responders*, *n = 291* *(multiple answers given)* *n (%)*
Excessive tiredness	217 (74.6)
Crying fits	104 (35.7)
Excessive anger	115 (39.5)
Lack of sleep	176 (60.5)
Depression	69 (23.7)
Weight changes	67 (23.0)
Need a psychologist	71 (24.4)
Get sick often	37 (12.7)
Other (backpain, social retirement…)	53 (18.2)
***How often do you go to the doctor?***	*Responders*, *n = 288* *n (%)*
Never	9 (3.1)
1/year	90 (31.3)
4–5 times/year	138 (47.9)
Often	45 (15.6)
Other	6 (2.1)
***Impact on your personal life***	*Responders*, *n = 277* *(multiple answers given)* *n (%)*
Interruption of personal activities (hobbies, travel…)	202 (72.9)
Separation of friends	130 (46.9)
Impact on family members	65 (23.4)
Impact on partner	56 (20.2)
None	3 (1.1)
Other	31 (11.1)
***What kind of support should be improved?***	*Responders*, *n = 447* *n (%)*
Presence of multidisciplinary team	384 (85.9)
Support for home assistance	303 (67.8)
Psychological support	246 (55.0)
Reference in case of need (telemedicine, etc.)	242 (54.1)
Presence of a neurologist	201 (45.0)
More support from general practitioner	196 (43.8)
Reimbursement	156 (34.9)
Temporary assistance	126 (28.2)
Home nursing service	121 (27.1)
Facilitation in transportation	118 (26.4)
Legal assistance	117 (26.2)
Sharing experiences	114 (25.5)
Other (web site, hospital/hotel, drugs delivery, job facilitation)	259 (57.9)

Three-hundred and sixty-one participants (75%) were women, and the majority (46%) had an age included between 55 and 70 years old. In this age range women were more represented than men (51% vs. 32%), whereas for the age range > 70 years, men were more than women (50% vs. 25%, respectively). Regarding education, most of the caregiver participants had a high school education (46%) or were graduated (36%). The most reported relationship with patients was “partner” (73%), and 21% of caregivers had a direct kinship (sister/brother or daughter/son) with the assisted relative. A large majority (79%) lived together with the patients. Almost 70% of caregivers had a duration of the assistance lasting from 1 year to 10 years and up to 24% assisted their relative patient for more than 10 years. The burden of assistance in terms of days spent with the patients was different over time. Indeed, the caregivers reported that in the first period of assistance 48% of participants spent 1–2 days a week, while with the progression of disease almost 74% of caregivers spent every day with the patients. The caregiving hourly commitment lasted from a couple of hours a day to a complete daily engagement in 42% of caregivers. The caregiving activities were related to outside accompaniment (81%), home care (65%), personal assistance (49%), administration of therapies (45%) and supervision (41%). More than half of our sample (51%) reported that they dealt with difficult situations related to specific aspects of assistance, but only 9% made a training for caregiving activities, and up to 40% thought that a training for assistance would be useful for the practical management of assistance activities, covering legal aspects and information on clinical referral centres.

**Fig. 1:**
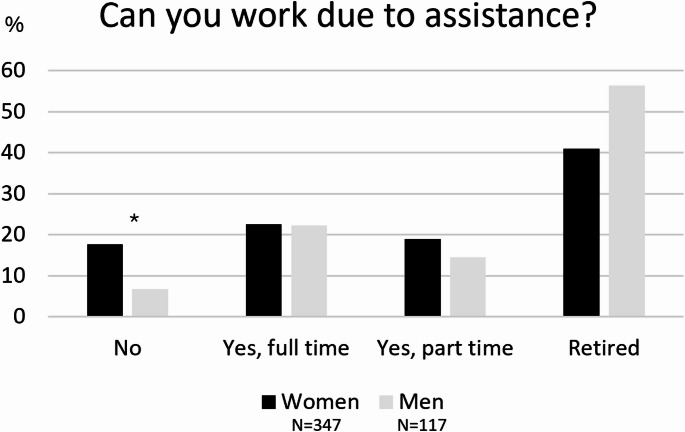
Work status features of women and men caregivers. * *p* < 0.05 for difference between women and men

Regarding the work status, 44% of the caregivers were retired, while 40% of the participants still worked (the majority were employees, 68%). Only 15%, mostly women, reported not working (Fig. 1). Among the worker caregivers, almost 70% reported at least 1 working day lost monthly due to caregiving activities and mostly of them (40%) reported 2–5 working days lost monthly. A slight majority of caregivers (54%) reported to have some facilitation at work for their caregiving, but 41% reported to not have access to working concession/facilitation.

Concerning the Economic Section, almost 68% of caregivers reported that they do not receive economic support, but only 18% asked for it. Only 63 participants (14%) reported the presence of an economic support, most including accompanying allowance and benefit from Law “104”, an Italian Law that allows work facilitations to those people who assist disabled patients. Almost 60% reported the presence of economic changes due to caregiving. The most reported causes for economic impact were the need of a rehabilitation treatment (62%), the need for help for house managing (53%), the presence of another caregiver (49%) and the need for travel for medical visit (41%).

**Fig. 2:**
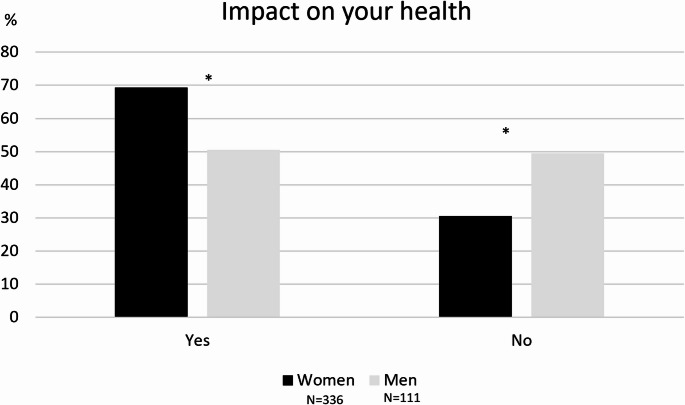
Health issues reported by women and men caregivers. * *p* < 0.05 for difference between women and men

Regarding the section Health and Personal Life, almost 65% of participants reported an impact on their health, mostly reported by women caregivers than men (69% vs. 50%, *p* < 0.001) (Fig. 2), in terms of “excessive tiredness” (74%), “lack of sleep” (60%) and other signs and symptoms of psychological stress, such as excessive anger (39%), depression (24%), and deserving a need for a psychologist in 24% of caregivers.

Finally, almost 86% of caregivers reported that the “presence of a multidisciplinary team” and “support for home assistance” (68%) should be improved in the care of PD patients.

### Participants reported information

Our caregiver participants reported information about 456 patients (see table S2 for details), the majority of whom were men (61%) with an age over 70 years old (58%) and retired (77%). The most reported disease was PD (97%), with a disease duration lasting between 4 and 7 years (27%), 8–12 years (26%), and 12–20 years (26%). In our sample, 51% of patients had a good autonomy, but 22% had a low level of self-sufficiency. Moreover, 51% of patients required a rehabilitation support, at home or in dedicated centers, and only 14% required house assistance.

## Discussion

In this nationwide study, we evaluated the caregiver burden of PD and the experiences of a large sample of Italian caregivers. To our knowledge, this is one of the largest nationwide studies using a semi-structured survey.

The survey was developed aiming at exploring the needs of Italian caregivers in order to understand how and what kind of future directions and strategies could be proposed for improving caregiver activities and decreasing caregiver burden. The reason why we developed an ad hoc instrument was the attempt to deepen the needs of the Italian caregiver, starting from the main determinants of caregiver burden present in literature including physical, emotional, social and financial domains. Specifically, Lesley and coll identified poor caregiver mental health as one of the main predictors of caregiver burden [5]. Alongside, several studies included some caregiving features as predictors of caregiver burden, such as caregiving duration, the presence of social support [9], working performances and productivity [12–14] together with clinical features of the assisted patients [18, 19].

Demographic characteristics of our sample were similar to those reported in previous Italian and international studies [11, 14, 18]. The profile of the typical caregiver identified by our survey, was a woman caregiver, with a most prevalent age of 55–70 years old and partner of the patient. The women caregivers reported greater difficulties in working activities and a higher impact on health due to assistance than men caregivers. The majority of patients assisted by responding caregivers were men (61%) with a most prevalent age over 70 years old and a disease duration distributed between 4 and 20 years. Usually, the assisted patient was older than the caregiver.

Previous studies identified the main determinants of caregiver burden, demonstrating that patients’ motor and non-motor symptoms affect the working performances of caregivers by reducing the time caregivers devoted to work, decreasing their productivity, and making their perceptions of quality of life more negative [12]. Additionally, it has been demonstrated that financial changes deriving from reduced productivity at work, also exerted a substantial influence in general burden associated with caregiving [19]. In our study, concerning the impact on work, 15% of caregivers reported that they could not work due to the assistance, and 76% reported an impact on work, in terms of decreasing working hours, asking for more smart working time and more rest days, changing job and also leaving job for a while.

Regarding the economic burden, it is well known that as the disease progresses, patients with PD become increasingly dependent on care, frequently provided by non-professional persons [4]. In our sample, most of the participants reported that they did not benefit from an economic support, and experienced economic changes due to caregiving activities and management and support of the assisted patients, especially for rehabilitation treatment and help for house managing. In this context, financial support for caregivers should include direct income payments and supplements, credits toward tax, public assistance, other social security programs, and grants to cover care-related activities and costs [4]. This is an important issue, and an effort should be done to promote novel interventions for improving optimization of treatment in the future, to enhance health outcomes and reducing the healthcare burden on people with PD, their caregivers, and society [3]. Thus, taking into account all these aspects, our study is in line with previous studies [3, 12, 19] considering difficulties in work activities as one of the principal domains affected by caregiver burden and influencing also the economic status of caregivers.

Another important topic is the need to be trained on activities related to assistance but also on financial and legal aspects. Among our caregivers, only 9% reported to have carried out a training on caregiver-related activities and it was evident the lack of specific training in the vast majority of caregivers. As previously demonstrated, the improvement of management and assistance through specific training strategies could potentially ensure better patient-centred communication and could reduce caregiver burden [20]. Concerning this aspect, the role of the institutions and association could be extremely relevant by promoting initiatives and enhancing awareness on caregiver burden within our sociocultural and healthcare framework. Indeed, as previously demonstrated, the improvement of management and assistance through specific training strategies could potentially ensure better patient-centred communication and could reduce caregiver burden [20].

One of the most important aspect and factor of caregiver burden is the impact on personal health and social activities. Almost 65% of our participants reported an impact on health in terms of “excessive tiredness”, “lack of sleep” and other symptoms encompassing depression, anxiety and anger, leading to the need of a psychologist, due to caregiving. Accordingly, previous studies [11, 18–20] showed that caregivers reported impaired levels of general well-being and psychosocial functioning, with an increased risk of psychiatric morbidity and persistent distress [14]. Thus, together with the above-mentioned lack of training on specific caregivers’ activities, also the lack of psychological support could contribute to the caregiver burden and decrease quality of life of caregiver.

Finally, the need of a multidisciplinary nature of the skills required in the care of PD patients clearly emerges as a resource capable to alleviate the caregiver burden in 86% of the answers. However, 68% of caregivers, required more support for home assistance. These aspects highlight further difficulties in the activity of caregivers and draw attention not only to the quality of the resources available, but also to the efficiency of the organization for the provision of support. In this scenario, future interventions to reduce the burden of PD caregivers could therefore be improved with the inclusion of mindfulness training programs.

### Gender-related differences

Studies found that demographic factors (such as ethnicity, gender and care-recipient diagnosis) accounted for a significant 12% of the variance in predicting caregivers’ gains [15]. Among our participants, some interesting gender differences came out. In our sample, age distribution among women and men caregivers was statistically significant, showing that women caregivers were younger than men caregivers. Concerning age, Vescovelli and coll [15] showed that this variable is one of the most significant predictors of caregiver burden, i.e. older caregivers reported more distress when compared to younger ones. Furthermore, in our sample women caregivers reported that they could not work due to the assistance more than men caregivers and experienced a greater impact on personal health than men caregivers. Accordingly, a recent review [21] showed that women caregivers usually report worse quality of life. Women care providers were twice as likely as male to report exhaustion and damage to their health resulting from care provision in PD, experiencing symptoms like anxiety and depression [22]. Additionally, in one recent qualitative study, women caregiver reported to have no active employment [23].

Considering all this data, our findings suggest the need for a multidisciplinary approach in the management of caregiver burden, mainly including the improvement of the economic aspect with facilitations and simplifications of administrative procedures, and the enhancement of psychological support for caregivers together with the improvement of specific training for caregiving activities.

This study presents some strengths and limitations. The main strength of our study was the extensive questionnaire, which encompassed several domains exploring all the possible aspects of caregiving activities and the large sample size, identifying our study as one of the largest nationwide studies on this topic. On the other hand, one of the main limits was the distribution of the survey that could not have reached all the interested people with the same coverage and the generalizability of data due to sampling-related problems, such as the use of a non-random sampling, the risk of selection bias together with people without internet access which might still not be reached or who are unwilling to participate in online surveys [24]. Another limit is the lack of validated tools, such as Zarit Burden Interview (BDI) [7]. Our survey is an exploratory fact-finding investigation on specific needs of caregivers of PD patients, who not only experienced a cognitive burden but also motor difficulties leading to a specific kind of assistance, allowing to recognize the real condition of a sample of caregiver of PD patients in Italy.

### Practical implications

Starting from these results, it is crucial to identify which aspects, potentially modifiable, may be most burdensome to allow interventions to better support PD patients and their caregivers. Determining the extent of caregiver burden may help healthcare professionals and researchers to find a strategy to improve specific interventions for this issue. Previous studies tried to identify strategies for addressing this issue by proposing models that could holistically manage caregivers financial, emotional and social needs, not just relieving the burden but supporting them to flourish [4]. In this context, the role of healthcare professionals together with scientific and patients’ associations such as “Fondazione LIMPE per il Parkinson ONLUS” and “Confederazione Parkinson Italia” could be to find strategies to:

1. ameliorate work facilitation and accelerate administrative practices;
2. improve economic support;
3. promote specific training dedicated to caregiving activities;
4. provide targeted psychological support for caregivers;
5. develop programs to furnish multidisciplinary approaches, especially at home for aiding caregivers in their daily assistance;
6. enhance awareness about the social aspect related to caregiver burden;
7. advocate the needs of caregivers at institutional level (Ministry of Health, Regional Health System) to provide specific strategies to decrease caregiver burden.

In conclusion, in this nationwide observational survey we provided information about the caregiver burden of PD in Italy, highlighting also peculiar gender-differences. Future research should be oriented to identify predictors of caregiver burden in order to decrease this aspect and to suggest specific approaches to address caregiver burden with customized support and resources.

## Electronic supplementary material

Below is the link to the electronic supplementary material.

Supplementary Material 1

## Data Availability

The datasets generated during and/or analysed during the current study are available from the corresponding author on reasonable request.
