# Supplementary material for: Caregiver burden in Parkinson’s disease: a nationwide observational survey
Source: Neurol Sci. 2025 Jun 23;46(10):5093–103. doi: 10.1007/s10072-025-08306-4 (PMC12488780; doi:10.1007/s10072-025-08306-4)
Supplement: Supplementary file 1 — Supplementary Material 1 [file 10072_2025_8306_MOESM1_ESM.docx]

**Table S1** Socio-demographics and clinical characteristics of patients

|  | **Total, n=456** |
| --- | --- |
| ***Gender, men (%)*** | 281 (61.6) |
| ***Age*** | *Responders, n=456*  *n (%)* |
| 26 – 55 years | 31 (6.8) |
| 55 - 70 years | 158 (34.6) |
| > 70 years | 267 (58.6) |
| ***Education*** | *Responders, n=458*  *n (%)* |
| None | 5 (1.1) |
| Elementary school | 45 (9.8) |
| Middle school | 90 (19.6) |
| High school | 190 (41.5) |
| Graduation | 128 (27.9) |
| ***Does he/she work?*** | *Responders, n=456*  *n (%)* |
| No | 48 (10.5) |
| Retired | 353 (77.4) |
| Yes, full time | 38 (8.3) |
| Yes, part time | 17 (3.7) |
| ***Working position*** | *Responders, n=55*  *n (%)* |
| Employee | 35 (63.6) |
| Freelance | 16 (29.1) |
| Farmer | 4 (7.3) |
| ***Diagnosis*** | *Responders, n=454*  *n (%)* |
| Parkinson’s Disease and Parkinsonisms | 442 (97.3) |
| Dementia | 4 (0.9) |
| Normal Pressure Hydrocephalus | 3 (0.7) |
| Other | 5 (1.1) |
| ***Disease duration*** | *Responders, n=456*  *n (%)* |
| < 3 years | 53 (11.6) |
| 4-7 years | 126 (27.6) |
| 8-12 years | 119 (26.1) |
| 12-20 years | 118 (25.9) |
| > 20 years | 40 (8.8) |
| ***Level of self-sufficiency*** | *Responders, n=454*  *n (%)* |
| High | 230 (50.7) |
| Mild | 122 (26.9) |
| Low | 102 (22.5) |
| ***Patients’ Association*** | *Responders, n=454*  *n (%)* |
| Yes | 257 (56.7) |
| No | 197 (43.3) |
| ***Why not*** | *Responders, n=185*  *n (%)* |
| Not renewed | 39 (21.1) |
| Not interested | 58 (31.3) |
| Not found | 88 (47.6) |
| ***Rehabilitation support*** | *Responders, n=431*  *n (%)* |
| No | 210 (48.7) |
| Yes, at home | 78 (18.1) |
| Yes, at a rehabilitation centre | 143 (33.2) |
| ***House assistance*** | *Responders, n=430*  *n (%)* |
| No | 368 (85.6) |
| Yes | 62 (14.4) |
|  |  |
